# Management of an Open Acetabular Fracture in a Skeletally Immature Patient

**DOI:** 10.2174/1874325000701010009

**Published:** 2007-11-20

**Authors:** Sarah Y Clutter, Steven J Morgan, Mark Erickson, Wade R Smith, Philip F Stahel

**Affiliations:** 1 Department of Orthopedic Surgery, Denver Health Medical Center, University of Colorado School of Medicine, Denver, CO 80204, USA; 2 Department of Pediatric Orthopedics, The Children’s Hospital, University of Colorado School of Medicine, Denver, CO 80218, USA

## Abstract

**Background:**

Open acetabular fractures in children are rare, but potentially devastating injuries. Secondary to the low incidence, there is an apparent lack of reports on appropriate management strategies for open pediatric acetabular fractures in the literature.

**Methods:**

Description of a case study.

**Results:**

A 3 years and ten months-old girl was ejected as a passenger from an all terrain vehicle. She sustained a displaced, grade IIIA open left anterior column acetabular fracture. The injury was treated by extending the open wound to a formal first window of the ilioinguinal approach. After surgical debridement, the anterior column was reduced anatomically and fixed by two lag screws which avoided the tri-radiate cartilage. A vaginal laceration was debrided and repaired. The patient was treated in a spica cast without weight bearing on the left lower extremity for 8 weeks. No perioperative complications occurred. The acetabular fracture healed in an anatomic position within 8 weeks. To avoid premature closure of the tri-radiate cartilage, the patient underwent a physeal bar resection at one year after injury. At two-year follow up, she was walking and running without pain and had a free range of motion of her left hip.

**Conclusions:**

Operative management should represent the therapy of choice for open, displaced pediatric acetabular fractures. After fracture healing, a scheduled physeal bar resection may be required for injuries which involve the tri-radiate cartilage.

## INTRODUCTION

Acetabular fractures are rare in childhood [1-3]. These injuries are mainly caused by high-energy trauma, since the force required to fracture the strong and elastic pelvis in children is significant [4-6]. Injury to the acetabulum in the skeletally immature patient can lead to long term sequelae with respect to future growth due to injury of the tri-radiate cartilage [7]. The age at time of injury and the extent of residual displacement appear to represent the “key” determinants for long-term outcome of pelvic and acetabular fractures in childhood [7,8]. Recently, the operative fixation of unstable pediatric pelvic and acetabular fractures was advocated to restore pelvic symmetry and periarticular anatomy [8,9]. This treatment modality was shown to be associated with favorable clinical results and a low incidence of perioperative complications [8-10].

Open fractures involving the acetabulum in pediatric patients are exceedingly rare [11]. We report the case of a young girl who sustained a grade IIIA open anterior column acetabular fracture and suggest a treatment modality for these rare injuries.

## CASE REPORT

A 3 years and ten months old unhelmeted girl who was riding as a passenger on an all terrain vehicle (ATV) was ejected from the back passenger position. The patient was evaluated and transported by first responders to the local hospital by ambulance. On arrival, the patient was alert and responsive. Vital signs showed a respiratory rate of 22/min, a heart rate of 100/min, and a blood pressure of 100/86 mmHg. Physical examination revealed an open wound of about 6 cm length over the left iliac crest, a vaginal laceration, and a deformity of the left clavicle. The a.p. chest radiograph showed signs of pulmonary contusion in the left hemithorax and a minimally displaced left midshaft clavicle fracture.

**Fig. (1):**
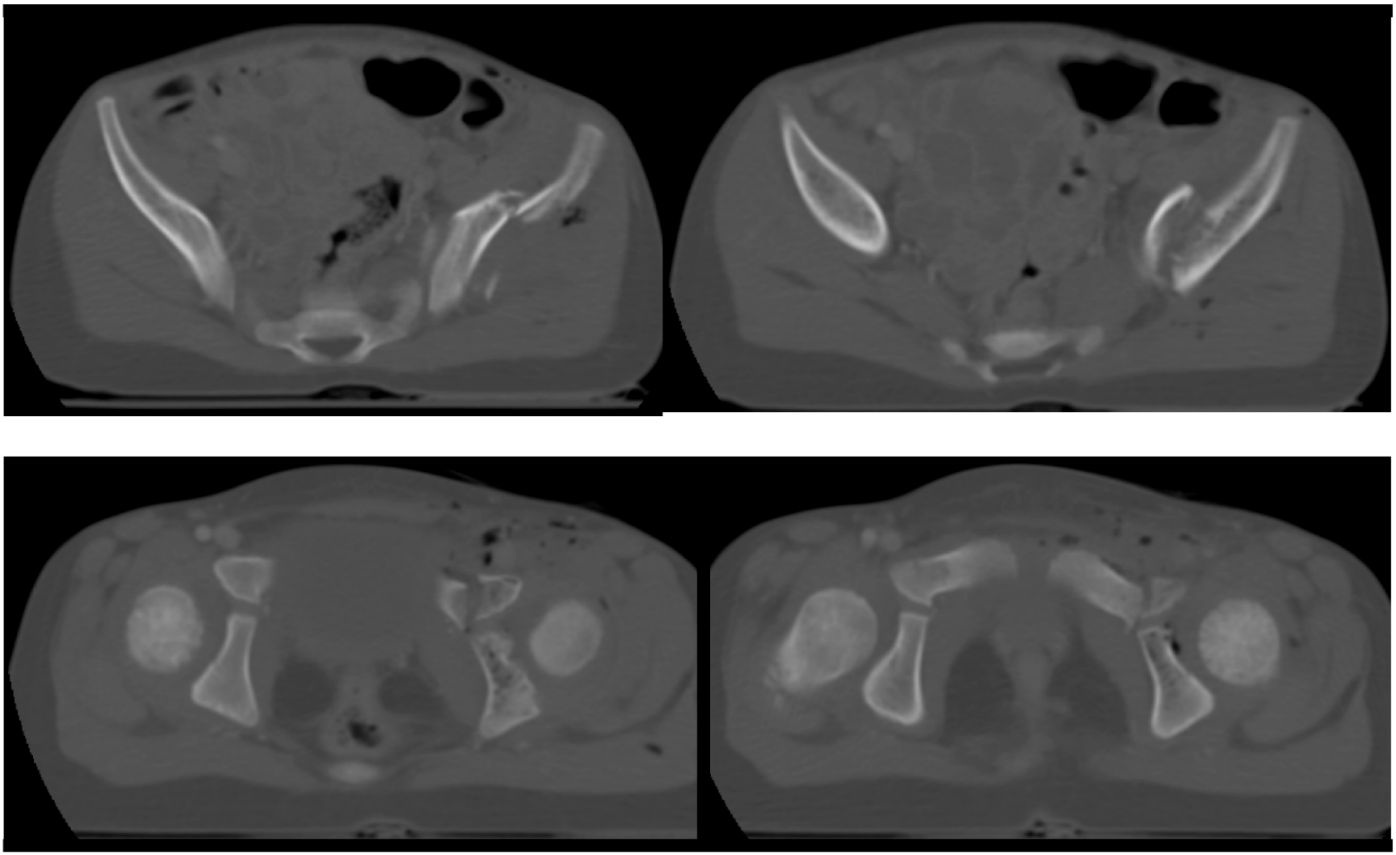
Axial CT scan at day of injury revealing a left anterior column acetabular fracture which involves the tri-radiate cartilage.

Initial laboratory studies indicated a hemoglobin of 9.8 g/dL and a hematocrit of 28%. Fluid resuscitation was initiated with 725 mL of 0.9% saline intravenously. The focussed assessment sonography for trauma (FAST) exam revealed a small amount of intraperitoneal free fluid. Since the patient was hemodynamically stable, an abdominal and pelvic CT scan was performed to objectify a potential bleeding source, due to the positive FAST exam. The CT studies revealed a grade I liver laceration and a left anterior column acetabular fracture which involved the tri-radiate cartilage (Fig. **1**). Based on this severe injury pattern, the girl was then transferred to our level 1 trauma center for definitive care. She was alert and hemodynamically stable throughout the transport.

Upon arrival in our emergency department, the girl’s respiration rate was 20/min, heart rate 162/min, and blood pressure 116/74 mmHg. Antibiotic prophylaxis was administered due to the open fracture (Ancef 400 mg i.v.) and a tetanus immunization was provided. Both lower extremities had normal and symmetric pulses, normal capillary refill times of 2 seconds, and a normal neurological exam. The patient was immediately taken to the OR for emergent debridement and fixation of the left open acetabular fracture and exploration and repair of the vaginal laceration under general anesthesia.

The anterior column acetabular fracture was exposed by extending the wound on the left iliac crest to a formal first window of the ilioinguinal Letournel approach. The open fracture and the surrounding soft tissues were debrided and irrigated. The anterior column acetabular fracture was reduced by manipulating the involved portion of the iliac wing with a *Faraboeuf *reduction forceps while internally rotating the anterior column with ball spike pusher placed at the level of the pelvic brim. The reduction was maintained with two 3.5 mm screws placed with lag fixation technique. Fluoroscopic guidance was used to insure that the fixation avoided the tri-radiate cartilage. The vaginal wall laceration was debrided and repaired by the urology service.

**Fig. (2):**
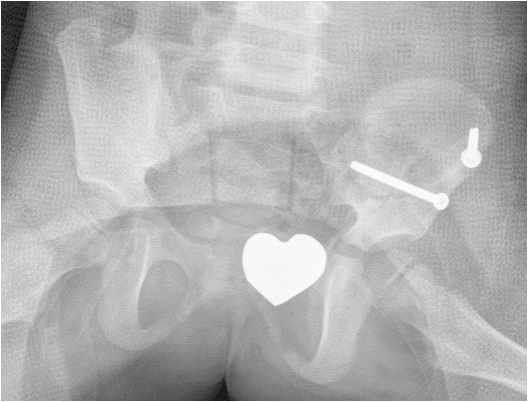
The postoperative pelvic X-ray in a spica cast shows anatomic reduction of the left acetabular fracture.

The patient was postoperatively monitored in the pediatric intensive care unit for 24 hours and remained hemodynamically stable. The left lower extremity showed a normal postoperative neurovascular exam. The left clavicle fracture was treated nonoperatively. The patient was placed in a left hip spica cast on day 2 to protect the acetabular repair (Fig. **2**). She was transferred to The Children’s Hospital in stable condition. She was kept non-weight bearing on the left leg.

**Fig. (3):**
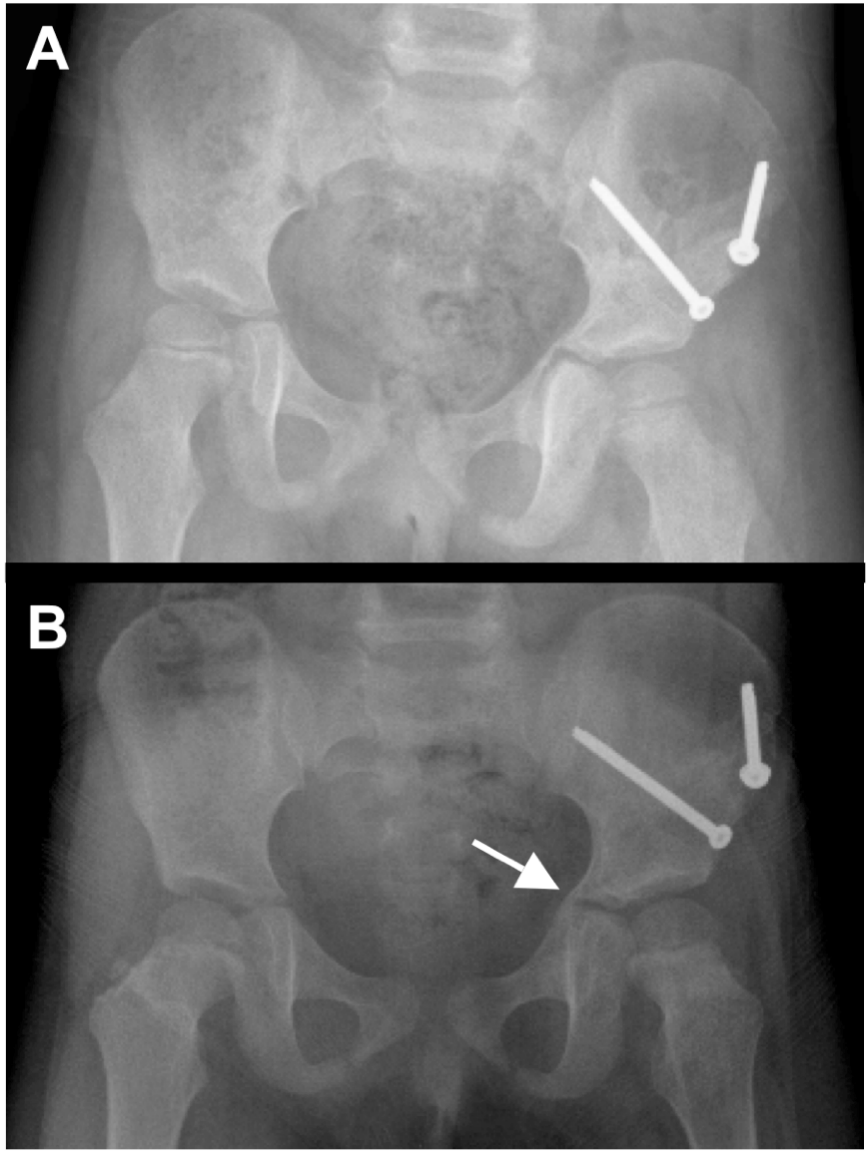
Follow-up X-rays taken at 8 weeks **(A)** and 4 months **(B)** demonstrate a healed left acetabular fracture in anatomic position and indicate an early physeal closure with callus formation about the tri-radiate cartilage (arrow in panel B).

**Fig. (4):**
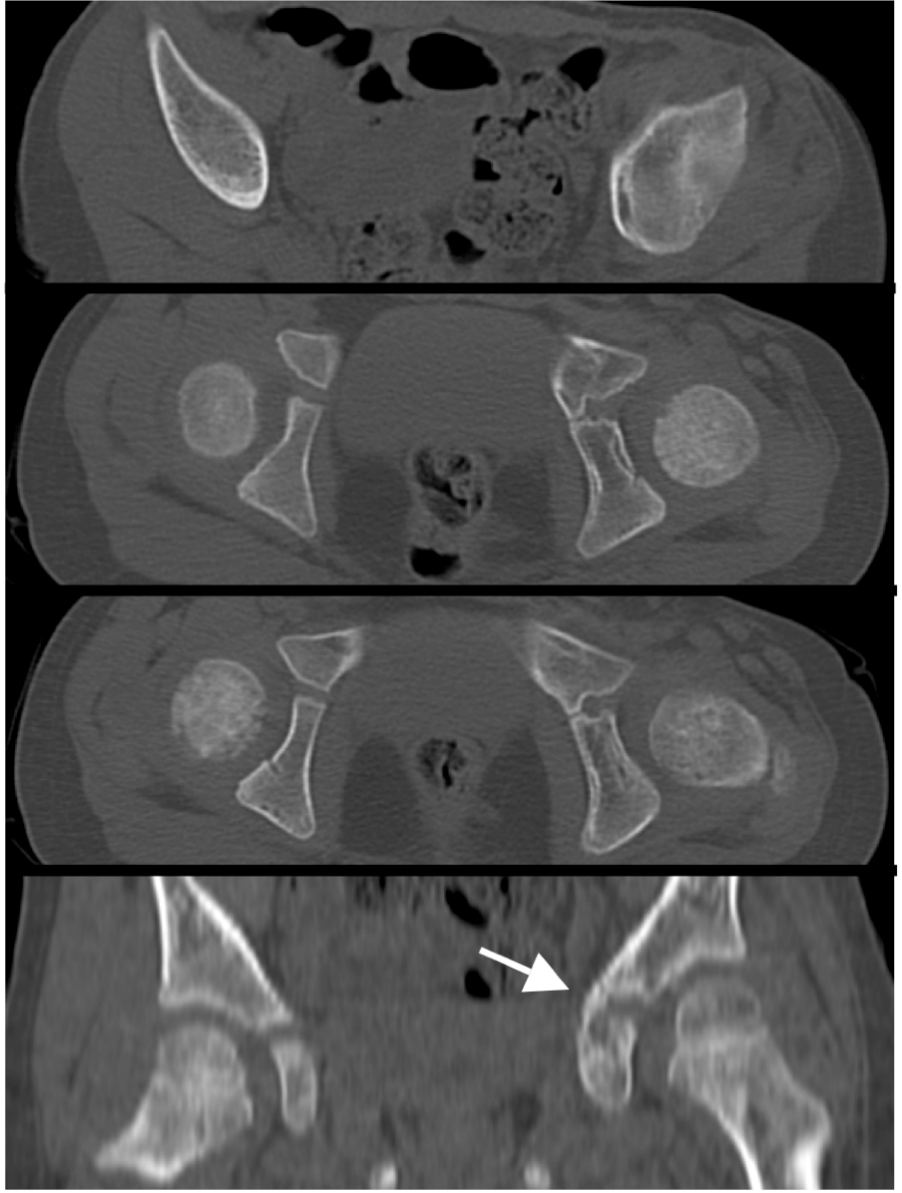
A CT scan obtained after hardware removal at 10 months demonstrates the healed fracture in anatomic position and confirms the suspected early physeal closure of the left acetabulum (arrow in the coronal view).

**Fig. (5):**
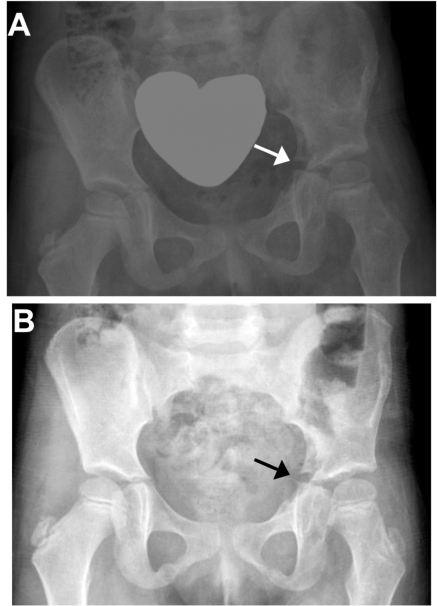
Pelvic X-ray at 4 months **(A)** and one year **(B)** after physeal bar resection shows a wide open spacing without signs of recurrent premature closure of the tri-radiate cartilage (arrows).

When the patient followed up in our orthopedic clinic at two weeks for change of spica cast and suture removal, the wounds had healed well without any signs of infection. Further follow-up visits were scheduled on a monthly basis. The spica cast was removed at 8 weeks. At this time, radiographs showed adequate fracture healing in an anatomic position (Fig. **3A**). The patient was subjectively progressing well, with no pain in her left hip or left clavicle. Radiographs taken at 4 months revealed callus formation about the tri-radiate cartilage (Fig. **3B**). Eight months after surgery, the patient was scheduled for elective hardware removal (two screws and two washers). A CT obtained at 10 months confirmed a suspected early physeal closure of the tri-radiate cartilage with a partial physeal arrest (Fig. **4**). Thus, the patient underwent physeal bar resection of the tri-radiate cartilage of the left hip one year after injury. At four months and one year after physeal bar resection, radiographs showed a wide-open spacing with no recurrence of the premature closure of the tri-radiate cartilage (Fig. **5**). At two year follow-up, the almost 6-year old girl was subjectively doing very well. She was walking and running without pain, had equal leg lengths and full range of motion of her left hip.

## DISCUSSION

Pediatric acetabular fractures are uncommon, with an annual incidence of about one case per 100,000 children [1,3,9,12]. Accordingly, the occurrence of open acetabular fractures in a skeletally immature individual is exceedingly rare [1,2,9]. Laceration of the perineum, vagina, and rectum should be considered potential sites for open fractures, thus warranting the use of direct or speculum exploration to prove otherwise [5,8]. Despite the infrequent nature of these injuries, pediatric pelvic trauma accounts for a disproportionate number of pediatric fatalities [5,8]. Along with pediatric pelvic fractures, acetabular fractures rank second to skull fractures associated with head injuries, with respect to morbidity and mortality [5,8,9,13] from musculoskeletal injury. Notably, open pediatric pelvic fractures are associated with a mortality of about 50% [5,8,9,13].

An important, potential sequelae of pediatric acetabular fractures is damage to the triradiate cartilage [7]. Trauma to the triradiate cartilage may adversely affect acetabular development which occurs from birth through adolescence [1,7]. Damage to the tri-radiate cartilage can result in premature closure and therefore the patient’s age at the time of injury and the extent of fracture displacement represent the most important factors affecting resultant deformity and disability [2,7,8]. In a child under 10 years of age, more severe dysplasia is seen in the long-term as opposed to adolescents of more than 10 years [6,7]. While affected patients may not complain of clinical symptoms for up to two decades after injury, they will eventually suffer from long-term sequelae including subjective signs of arthritis with decreased range of motion of the affected hip, pain and positive Trendelenburg sign [2,7].

A search of three online databases (Pubmed, Cochrane, Ovid/Medline) revealed only two published articles in English language on the topic [4,11] and none specifically dealing with open acetabular fractures. One paper analyzed the diagnostic role of MRI imaging for pediatric posterior wall acetabular fractures in the setting of traumatic hip dislocation [4]. The second paper describes a retrospective analysis of 166 pediatric pelvic fractures, of which six involved the acetabulum in skeletally immature patients [11]. None of these fractures were treated surgically by open reduction and internal fixation [11].

In the present paper, we report for the first time, to our knowledge, on the management of an open pediatric acetabular fracture. Our treatment was predicated upon the basic surgical principles of acutely preventing infection and stabilizing the bone and soft tissues in the presence of open fracture. The early operative management by surgical debridement, anatomic reduction and fixation with primary wound closure and repair of the vaginal laceration led to an excellent outcome at 2 years of followup. Since the patient was a 3 years old girl at the time of injury, she had an expected 10 to 12 more years of growth on average [11]. She underwent a physeal bar resection one year after trauma due to radiographic signs of a physeal arrest. The patient was asymptomatic and has a normal range of motion of her left hip two years after injury. She will be monitored closely by clinical and radiographic follow-ups as she continues to grow. In summary, we recommend a surgical debridement and an open anatomic reduction and internal fixation of displaced open pediatric acetabular fractures as the therapy of choice in hemodynamically stable patients.
